# The Rice Floral Repressor *Early flowering1* Affects Spikelet Fertility By Modulating Gibberellin Signaling

**DOI:** 10.1186/s12284-015-0058-1

**Published:** 2015-07-24

**Authors:** Choon-Tak Kwon, Suk-Hwan Kim, Dami Kim, Nam-Chon Paek

**Affiliations:** Department of Plant Science, Plant Genomics and Breeding Institute, and Research Institute of Agriculture and Life Sciences, Seoul National University, Seoul, 151-921 Republic of Korea; Crop Biotechnology Institute, GreenBio Science and Technology, Seoul National University, Pyeongchang, 232-916 Republic of Korea

**Keywords:** Rice, *Early flowering1*, Spikelet fertility, Seed setting rate, Anther development, Pollen viability, GA signaling

## Abstract

**Background:**

Gibberellic acid (GA; or gibberellin) affects the development of floral organs, especially anthers and pollen, and perturbation of development of male floral organs can cause sterility. Many studies of GA signaling have concentrated on anther development, but the effect of GA on grain production remains to be examined.

**Results:**

Using a cross of ‘Milyang23 (M23)’, which has a functional allele of *Early flowering1* (*EL1*), and ‘H143’, which has a nonfunctional *el1* allele, we generated heterogeneous inbred family-near isogenic lines (HNILs) that are homozygous for *EL1* [HNIL(M23)] or *el1* [HNIL(H143)]. Here, we found that HNIL(H143) exhibited anther deformities and low pollen viability. The expression of *GAMYB*, a major activator of GA signaling, and its downstream genes *CYP703A3* and *KAR*, mainly involved in pollen formation, increased abnormally during spikelet development; this activation of GA signaling may cause the sterility. To confirm the negative effect of the *el1* mutation on spikelet fertility, we examined a line carrying a T-DNA insertion *el1* mutant [hereafter ZH11(*el1*)] and its parental cultivar ‘Zhonghua11 (ZH11)’. ZH11(*el1*) showed nearly identical defects in anther development and pollen viability as HNIL(H143), leading to decreased seed setting rate. However, the elite *japonica* cultivar Koshihikari, which has a nonfunctional *el1* allele for early flowering in long days, produces fertile spikelets and normal grain yields, like other elite *japonica* cultivars. This indicates that as-yet-unknown regulator(s) that can overcome the male sterile phenotype of the *el1* mutation must have been introduced into Koshihikari.

**Conclusions:**

The *el1* mutation contributes to early flowering in *japonica* rice under long days but fails to limit GA signaling, thus negatively affecting spikelet fertility, which results in a loss of grain yield. Thus, *EL1* is essential for photoperiod sensitivity in flowering as well as spikelet fertility in grain production.

**Electronic supplementary material:**

The online version of this article (doi:10.1186/s12284-015-0058-1) contains supplementary material, which is available to authorized users.

## Background

Rice (*Oryza sativa* L.) is a major staple food for more than a half of the world population, mainly in Asia. Enhancing the production of rice will require improvements of the major yield components, such as the number of panicles per plant, the number of spikelets per panicle, the seed setting rate, and the weight of each grain. Furthermore, these improvements must maintain the balance of yield-related traits; for example, inordinate tillering often decreases the grain yield per plant, because late-producing, infertile tillers compete for resources with the main, fertile shoot, thus decreasing spikelet fertility or seed setting rate (Liu et al. 2013).

Spikelet fertility, an important component of grain production, is influenced by environmental conditions and genetic background. Anther development and pollen viability particularly affect spikelet fertility (Liu et al. 2013), and numerous genetic studies have shown a significant relationship between the expression of the genes controlling the development of anthers and pollen and the fertility of the spikelet. For instance, normal anther formation and dehiscence in rice require *ANTHER INDEHISCENCE1* (*AID1*) and the SUMO E3 ligase encoded by *OsSIZ1* (Zhu et al. 2004; Thangasamy et al. 2011).

Moreover, phytohormones and the genes involved in hormone biosynthesis or signal pathways affect anther dehiscence and pollen maturation. In Arabidopsis (*Arabidopsis thaliana*), auxin and jasmonic acid have crucial functions in fertility, acting by regulating anther and pollen development (Park et al. 2002; Cecchetti et al. 2008). Gibberellic acid (GA; also gibberellin) also has important roles in germination and floral organ development, acting by regulating stamen and anther formation (Nester and Zeevaart 1988; Ritchie and Gilroy 1998; Goto and Pharis 1999; Woodger et al. 2003). Moreover, GA is critical for pollen formation and pollen tube growth (Singh et al. 2002; Chhun et al. 2007), and GA signaling pathways are conserved between rice and Arabidopsis (Hedden and Phillips 2000; Fleet and Sun 2005). In particular, the homology of several factors involved in GA signaling and the strong similarities in stamen development between Arabidopsis and rice indicate the existence of conserved pathways of anther and pollen development within angiosperms (Chen et al. 2005; Itoh et al. 2005b; Wilson and Zhang 2009; Plackett et al. 2011).

Phenotypic screens, such as those for GA-insensitive dwarf or slender-type mutants in Arabidopsis and rice, have identified several genes necessary for GA-responsive signaling (Aya et al. 2009). The identification and functional analysis of the mutated genes has improved our understanding and allowed the construction of a model of GA signal transduction (Ueguchi-Tanaka et al. 2007b). In GA signal transduction in rice, the biologically active GA_4_ directly binds to the soluble GA receptor GA INSENSITIVE DWARF1 (GID1) (Ueguchi-Tanaka et al. 2005) and the GA_4_-GID1 complex physically interacts with the DELLA protein SLENDER RICE1 (SLR1) (Ueguchi-Tanaka et al. 2007a), leading to degradation of SLR1 through the SCF^GID2^ complex in the 26S proteasome pathway (Sasaki et al. 2003; Gomi et al. 2004). SLR1 controls GA signaling by negatively regulating the expression of GA-responsive genes such as *GAMYB* (Ikeda et al. 2001; Itoh et al. 2002; Aya et al. 2009). The central negative regulator of GA signaling, SLR1 exists in phosphorylated and non-phosphorylated forms in vivo (Sasaki et al. 2003; Gomi et al. 2004; Itoh et al. 2005a). Early work proposed that the degradation of SLR1 by the SCF^GID2^ complex depends on its phosphorylation (Sasaki et al. 2003; Gomi et al. 2004). However, subsequent work showed that non-phosphorylated SLR1 interacts with GID2, indicating that the phosphorylation of SLR1 is not essential for its degradation (Itoh et al. 2005a).

Several reports have described the post-translational modification of SLR1 protein, but few reports have examined its modifiers. Rice *SPINDLY*, encoding an O-linked N-acetylglucosamine transferase, does not control the stability of SLR1 but probably participates in the repression of SLR1 activity in GA signaling (Shimada et al. 2006). *Early flowering1* (*EL1*), encoding casein kinase I (CKI), is associated with the negative regulation of GA signaling, acting by phosphorylating SLR1 protein and activating its function in repressing gene expression (Dai and Xue 2010). Although the phosphorylation of SLR1 by EL1/CKI contributes to maintaining SLR1 protein stability and activity (Dai and Xue 2010), the function of phosphorylated SLR1 protein still remains unclear.

The degradation of DELLA proteins caused by perception of GA triggers the transcription of downstream genes involved in GA signaling (Itoh et al. 2002; Murray et al. 2003; Ueguchi-Tanaka et al. 2007b). GAMYB, the main transcription factor that initiates GA signaling, acts as a positive transcriptional regulator of the α-amylase gene in the cereal aleurone during germination (Gubler et al. 1995; Gubler et al. 1999; Gubler et al. 2002; Kaneko et al. 2004). A little over a decade ago, several reports showed that GAMYB is also essential for anther and pollen development in angiosperms (Murray et al. 2003; Achard et al. 2004; Kaneko et al. 2004; Tsuji et al. 2006; Chhun et al. 2007; Aya et al. 2009). For example, the knockout mutants of *GAMYB* in rice showed a severe defect in spikelet development, particularly in anther and pollen development (Kaneko et al. 2004).

GAMYB functions as a transcription factor, activating the expression of its downstream target genes regulating the exine and Ubisch body formation in pollen by directly binding to their promoters (Aya et al. 2009). Overexpression of *GAMYB* in barley (*Hordeum vulgare*) causes abnormal anthers with defective dehiscence, reduced length, and lighter color (Murray et al. 2003). In Arabidopsis, the *GAMYB*-like genes *MYB33* and *MYB65* contribute to anther development, similar to the role of *GAMYB* in rice and barley (Millar and Gubler 2005). These results demonstrate that *GAMYB* participates in GA signaling, which strongly affects floral development of angiosperms, mainly in anther and pollen growth.

The formation of male reproductive organs in plants requires numerous developmental steps including microspore maturation, meiosis, sporogenous cell differentiation, and stamen specification (McCormick 2004; Scott et al. 2004; Ma 2005; Guo and Liu 2012). Various investigations of the transcriptome identified many transcripts that are expressed in each developmental stage of anther and male gametophyte development in rice (reviewed by Guo and Liu 2012). Furthermore, some transcriptional analyses suggested that GA signal transduction occurs in developing microspores and the tapetum within the anther (Hirano et al. 2008; Hu et al. 2008). Microarray and comprehensive network analyses of the expressed genes in the rice anther have identified some genes in the GA signaling pathway, such as *CYP703A3* and *KAR*, which encode a cytochrome P450 hydroxylase and β-ketoacyl reductase, respectively (Aya et al. 2009; Aya et al. 2011). This finding strongly suggests that GA is involved in the control of anther and pollen development (Kaneko et al. 2004; Chhun et al. 2007; Aya et al. 2009) and also indicates that GA is strongly associated with male sterility and spikelet fertility. Although recent investigations of GA signaling have focused on the consequences of GA deficiency in stamen development, over-activation of GA signaling also negatively affects silique fertility in Arabidopsis (Plackett et al. 2014).

In our previous study, we isolated heterogeneous inbred family-near isogenic lines (HNILs) from a F_7_ recombinant inbred line (RIL) heterozygous for the functional *EL1* allele and the nonfunctional *el1* allele (Kwon et al. 2014). The natural missense mutation in *el1* promotes flowering under non-inductive long-day conditions due to a defect in the protein kinase activity (Kwon et al. 2014, 2015). In this study, we found that the HNILs possessing *EL1* or *el1* showed different inflorescence and spikelet fertility phenotypes. Our results suggest that a loss of EL1/CKI activity causes excessive GA responses, resulting in abnormal development of male floral organs, finally resulting in decreased grain yield. Also, we discuss how the elite *japonica* cultivar Koshihikari, which harbors an *el1* allele, could have normal GA responses and normal fertility.

## Results

### HNIL(H143) Plants Have Deformed Anther Structure And Decreased Pollen Viability

GA participates in various developmental processes in plants, including floral organ development (Cheng et al. 2004; Aya et al. 2009). EL1 phosphorylates SLR1, a major repressor of GA signaling (Dai and Xue 2010), and *slr1* mutants are sterile (Ikeda et al. 2001). Previous studies reported that two types of natural missense mutations (types 2 and 3) in *EL1* occur in the world rice collection and these mutations lead to compromised kinase activity of SLR1. HNIL(H143), harboring a type-2 *el1* mutation derived from the ‘H143’ cultivar, shows an early-flowering phenotype only in long days, but not in short days, compared with the HNIL(M23), which has a functional *EL1* allele derived from the ‘Milyang23 (M23)’ cultivar (Kwon et al. 2014).

**Fig. 1:**
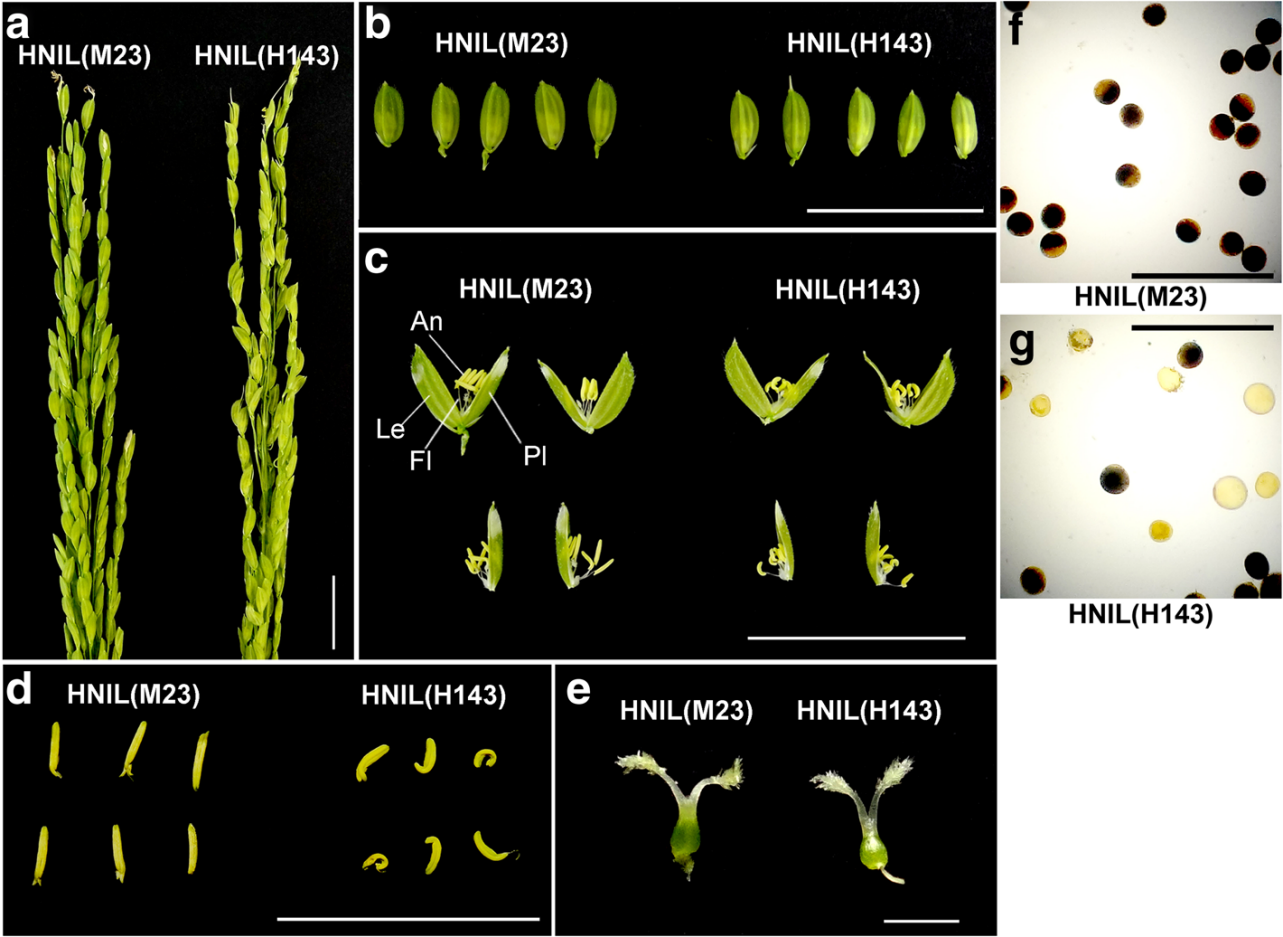
HNIL(H143) showed abnormal anther and pollen development compared with HNIL(M23). **a** Panicle structure of HNIL(M23) and HNIL(H143) plants at the heading stage. Scale bar = 20 mm. **b** Spikelets of the HNIL(M23) and HNIL(H143) plants. Scale bar = 20 mm (**b** and **c**). **c** Flowers of the HNIL(M23) and HNIL(H143) plants. An, anther; Le, lemma; Fl, filament; Pl, palea. **d** Anthers of the HNIL(M23) and HNIL(H143) plants. Scale bar = 10 mm. **e** Pistils of the HNIL(M23) and HNIL(H143) plants. Scale bar = 1 mm. Pollen grains from (**f**) HNIL(M23) and (**g**) HNIL(H143) plants. Pollen stained with I_2_-KI solution. Scale bar = 200 μm (**f** and **g**). The data represent five independent biological replicates

While conducting flowering-time experiments, we observed a severe decrease in the seed setting rate of HNIL(H143) plants in field conditions. To explore the possible effects of the *el1* mutation on rice fertility, we scrutinized the external and internal structure of spikelets of HNIL(M23) and HNIL(H143) plants at heading stage. The spikelets of HNIL(H143) showed normal structure (Fig. 1a and b), but the anthers of HNIL(H143) were shrunken and shorter than those of HNIL(M23), which showed normal structure (Fig. 1c and d). The pistils developed properly in the spikelets of HNIL(H143) (Fig. 1e). To examine the pollen viability in the shrunken anthers of HNIL(H143), we stained each anther using iodine-potassium iodide (I_2_-KI) solution, which revealed that HNIL(H143) pollen had lower viability than HNIL(M23) pollen (Fig. 1f and g). We also found that the pollen viability of the T-DNA insertion *el1* knockout mutant [hereafter ZH11(*el1*)] was also reduced compared to its parental cultivar, ‘Zhonghua11 (ZH11)’ (Additional file 1: Figure S1). Finally, we examined the spikelets of ‘H143’ and ‘M23’, which are the parental cultivars for the HNILs (Additional file 2: Figure S2). We also found that the H143 plants have shrunken anthers, similar to HNIL(H143) (Additional file 2: Figure S2). The H143 plants had fewer mature pollen grains than the M23 plants and many of the pollen grains in H143 were poorly stained by I_2_- KI solution (Additional file 2: Figure S2). Taken together, these results suggest that the shrunken anther structure and reduced pollen viability of HNIL(H143) are caused by the *el1* mutation in H143.

### HNIL(H143) Spikelets Showed Up-Regulated Expression Of *GAMYB* And Genes Related To Exine Formation At The Heading Stage

SLR1, phosphorylated and activated by EL1/CKI, represses the expression of the GA-responsive gene *GAMYB*, which encodes a transcription factor that positively regulates the α-amylase gene in aleurone cells (Itoh et al. 2002; Ueguchi-Tanaka et al. 2007b; Dai and Xue 2010). Loss of SLR1 function in rice induces a sterile phenotype, like mutants of its orthologue *slender1* (*sln1*) in barley (Lanahan and Ho 1988; Ikeda et al. 2001). *GAMYB* is also closely associated with anther development, acting through GA signaling to regulate several genes related to exine formation (Kaneko et al. 2004; Aya et al. 2009). In particular, transgenic barley plants overexpressing *HvGAMYB* showed a reduction in anther size and increased male sterility (Murray et al. 2003).

**Fig. 2:**
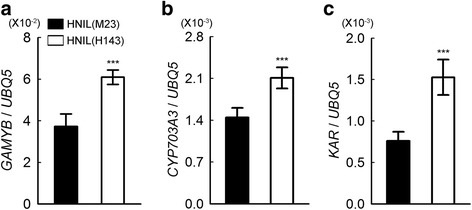
Up-regulation of *GAMYB* and pollen formation-related genes in the spikelets of HNIL(H143). Relative transcript levels of *GAMYB* (**a**), *CYP703A3* (*Cytochrome P450 hydroxylase*) (**b**), *KAR* (β*-ketoacyl reductase*) (**c**) in HNIL(M23) and HNIL(H143) spikelets were normalized to the transcript levels of *UBQ5*. The RT-qPCR was performed with total RNA from spikelets at heading stage. The data were obtained from three independent biological replicates. Reactions were repeated at least twice. Student’s *t*-test was used for statistical analysis (***P < 0.001). Means and standard deviations are recorded as values and vertical bars, respectively

We examined the transcript levels of *GAMYB* in the spikelets in HNIL(M23) and HNIL(H143) at heading stage. We found that *GAMYB* expression was up-regulated in HNIL(H143) compared to HNIL(M23) (Fig. 2a). Moreover, transcript levels of the exine formation-related genes *CYP703A3* and *KAR*, which are activated by GAMYB, were also up-regulated in HNIL(H143) (Fig. 2b and c). This result strongly suggests that abnormal up-regulation of the three GA signaling-related genes may cause the deformity of anthers in HNIL(H143).

### HNIL(H143) Plants Showed Over-Activation Of GA Signaling

EL1/CKI phosphorylates and stabilizes SLR1, which decreases the response to GA signaling. Thus, ZH11(*el1*) plants exhibit increased GA signaling response, even in the absence of GA or in the presence of uniconazole, an inhibitor of GA biosynthesis, or ABA; the *el1* seeds also display much higher α-amylase activity (Dai and Xue 2010).

**Fig. 3:**
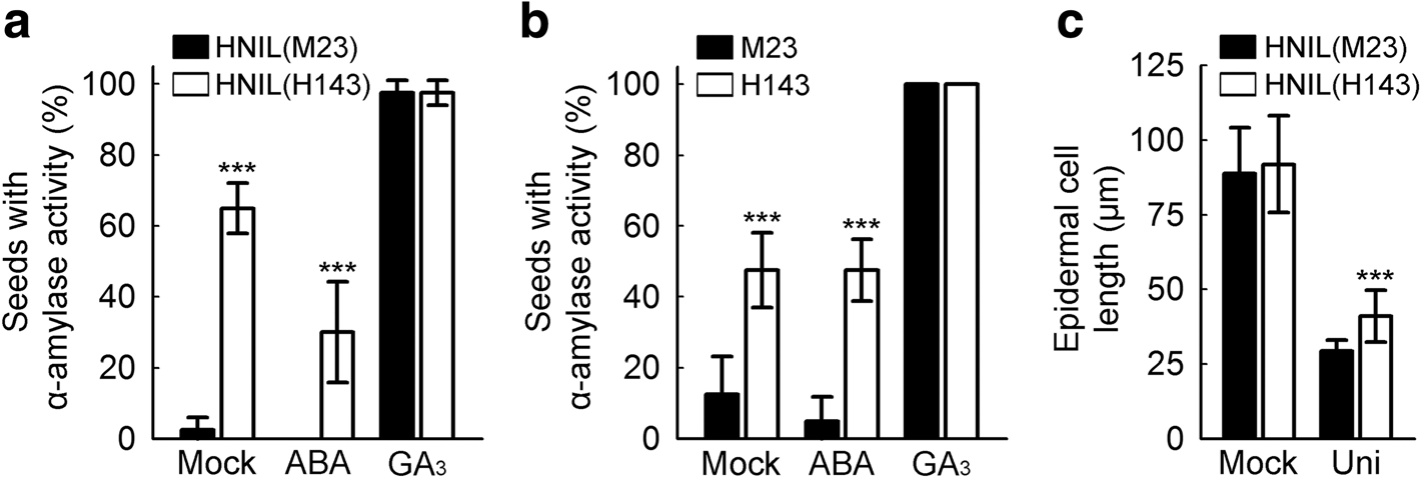
GA responses were enhanced in the seeds carrying the *el1*(H143) allele. Assays of α-amylase activity in the seeds of HNIL(M23) and HNIL(H143) (**a**) and M23 and H143 (**b**) on plates containing starch. The seeds were incubated on plates containing ABA (10 μM) or GA_3_ (1 mM) for 3 days at 28 °C. The numbers of seeds with α-amylase activity were compared by Student’s *t*-test (***P < 0.001, n = 40). This experiment was repeated at least 3 times. **c** Length of the epidermal cells in the 2nd leaf sheath of HNIL(M23) and HNIL(H143) plants. The plants were treated with uniconazole (Uni; GA biosynthesis inhibitor) for 1 day and grown in a growth chamber for 7 days. Student’s *t*-test was used for statistical analysis (***P < 0.001, n = 10)

For the functional analysis of the natural *el1* mutant allele in these HNILs (type 2; Kwon et al. 2014), we examined the α-amylase activity of mature seeds of HNIL(M23) and HNIL(H143), and their parents M23 and H143. Compared with HNIL(M23), the imbibed seeds of HNIL(H143) had remarkably elevated α-amylase activity in the absence of GA; the inhibitory effect of ABA on the α-amylase activity was also significantly alleviated (Fig. 3a and Additional file 3: Figure S3). The seeds of H143 showed a similar, enhanced α-amylase activity, compared with M23 (Fig. 3b and Additional file 3: Figure S3). Following treatment with exogenous GA_3_, almost 100 % of the seeds of both genotypes induced α-amylase activity (Fig. 3a and b).

GA stimulates the internode elongation and plant height. ZH11(*el1*) also had much longer epidermal cells in the leaf sheath than WT after uniconazole treatment, likely resulting from the increased GA response in ZH11(*el1*) plants (Dai and Xue 2010). We also observed that the epidermal cells of the 2nd leaf sheath in HNIL(H143) were significantly longer than those in HNIL(M23) after uniconazole treatment (Fig. 3c), supporting the notion that the *el1* mutation (type 2) in H143 leads to an enhanced GA signaling response, even in the absence of GA.

### Loss Of EL1 Function Decreased Yield-Related Traits By Reducing Spikelet Fertility

**Fig. 4:**
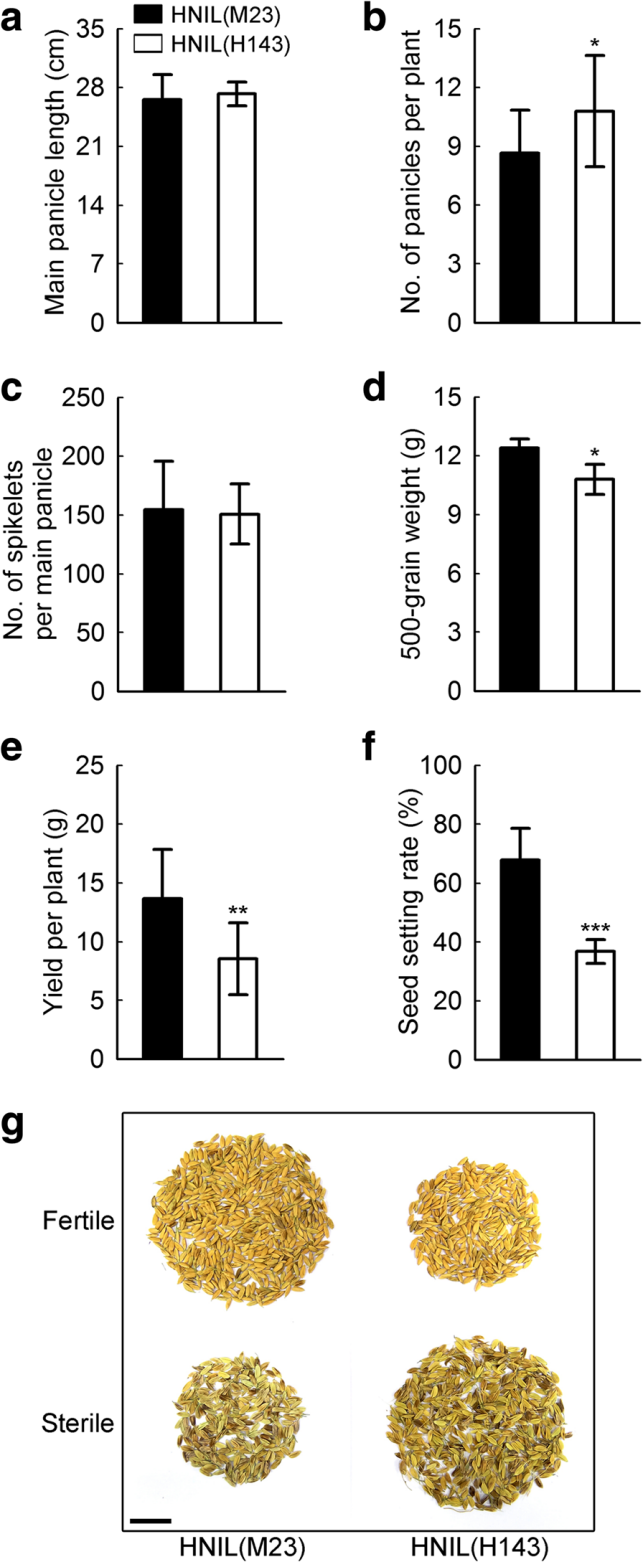
Yield-related agronomic traits were remarkably lower in HNIL(H143) compared with HNIL(M23). Agronomic traits were measured in HNIL(M23) and HNIL(H143) grown under natural long days in 2013. The measured traits were: main panicle length (**a**), number of panicles per plant (**b**), number of spikelets per main panicle (**c**), 500-grain weight (**d**), yield per plant (**e**), and seed setting rate (**f**). Twenty plants were used to measure each trait. Student’s *t*-test was used for statistical analysis (*P < 0.05, **P < 0.01, ***P < 0.001). Means and standard deviations are marked as values and vertical bars, respectively. (**g**) Fertile and sterile seeds from the whole plants of HNIL(M23) and HNIL(H143). Scale bar = 2 cm

To examine the effect of the *el1* mutation in H143 on grain production, we measured yield and yield components in HNIL(M23) and HNIL(H143) plants grown under natural long days in the paddy field in 2012 and 2013 (Fig. 4 and Additional file 4: Figure S4). We measured main panicle length, number of panicles per plant, number of spikelets per main panicle, 500-grain weight, grain yield per plant, and seed setting rate. Among these six agronomic traits, three major traits (500-grain weight, yield per plant, and seed setting rate) were significantly reduced in HNIL(H143) (Fig. 4d and f) and the others did not show any difference between HNIL(M23) and HNIL(H143), although the panicle number per plant in HNIL(H143) was slightly higher (Fig. 4a-c). The decrease in these three yield-related traits in HNIL(H143) was observed in both years (Additional file 4: Figure S4), suggesting that dramatic reduction of seed setting rate in HNIL(H143) is the main reason for the reduction of grain yield. This indicates that *EL1* function is essential for sustaining grain production, in addition to delaying flowering time in non-inductive long days (Kwon et al. 2014).

Next, we examined the yield and yield components of H143 and M23, the parental cultivars of HNIL(M23) and HNIL(H143) (Additional file 5: Figure S5). Most yield-related traits were lower in H143 than in M23, except the number of panicles per plant. Consistent with the disparity of seed setting rate in HNIL(M23) and HNIL(H143), the spikelet fertility of H143 was much lower than that of M23, which appears to be a main reason for the decrease in yield per plant. Together, these data suggest that the reduced fertility in HNIL(H143) was influenced by the natural allele of *el1* inherited from H143.

Finally, we examined the yield-related traits of ZH11(*el1*) corresponding to HNIL(H143) by comparing with those of its parental cultivar ZH11 corresponding to HNIL(M23) (Additional file 6: Figure S6). This comparison revealed that the seed setting rate of ZH11(*el1*) was about 12 % lower than that of ZH11 (Additional file 6: Figure S6). Taking all of these results together, we concluded that *EL1* is essential for high yield potential, especially for maintaining spikelet fertility.

### Koshihikari, An Elite *Japonica* Cultivar Carrying An *el1 * Mutation, Has No Defect In GA Signaling Or Spikelet Fertility

**Fig. 5:**
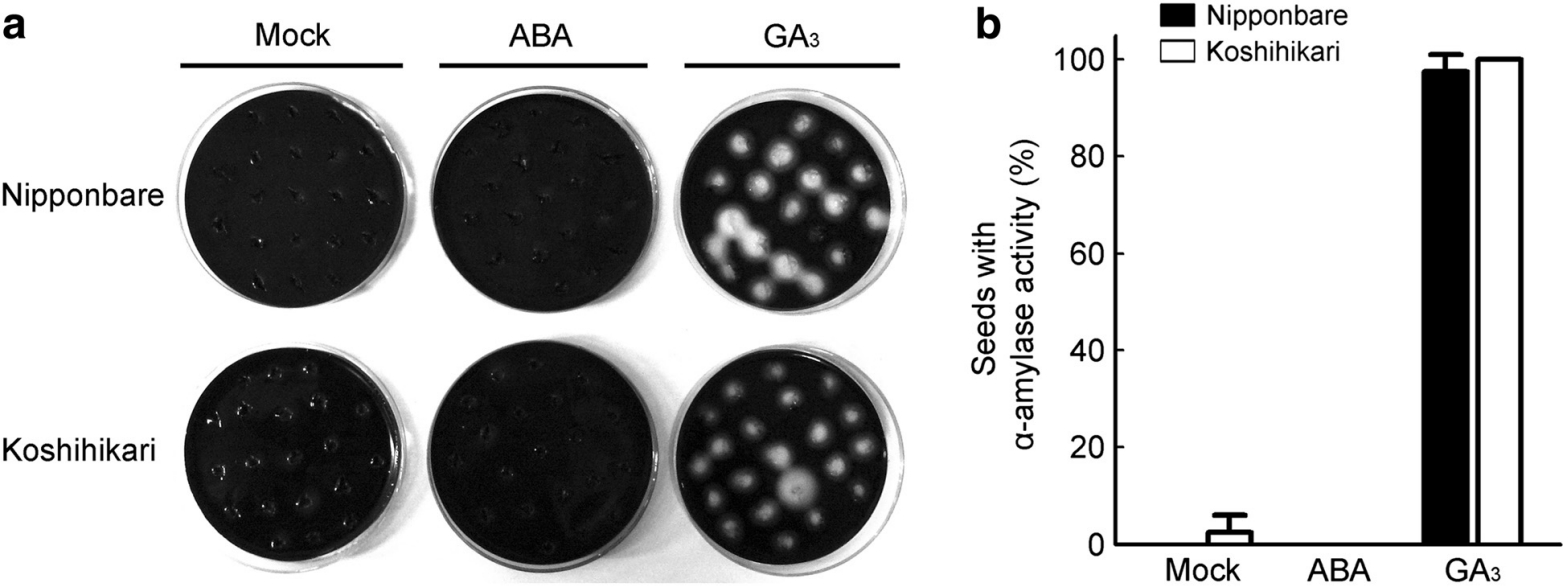
The α-amylase activities of Nipponbare and Koshihikari seeds. **a** The α-amylase in the endosperm of Nipponbare and Koshihikari seeds degrades the starch in the agar plates and thus can be detected as white spots in a dark background after the plates are stained with 0.1 % iodine and 1 % potassium iodide solution for 3 days at 28 °C. In addition to starch, the plates also contained 10 μM ABA and 1 mM GA_3_. **b** The graph of α-amylase activity in Nipponbare and Koshihikari (n = 40). This experiment was repeated at least 3 times

**Fig. 6:**
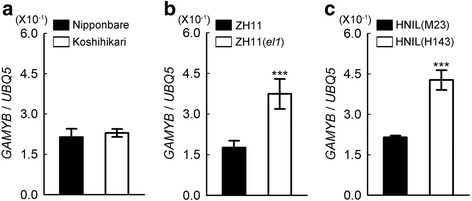
The levels of *GAMYB* transcript were not upregulated in Koshihikari. Relative transcript levels of *GAMYB* in Nipponbare and Koshihikari (**a**), ZH11 and ZH11(*el1*) (**b**), and HNIL(M23) and HNIL(H143) (**c**), were normalized to the transcript levels of *UBQ5*. The RT-qPCR was performed with total RNA from germinating seeds 2 days after soaking. Means and standard deviations were obtained from three replications. The experiments were repeated twice with similar results and Student’s *t*-test was used for statistical analysis (***P < 0.001)

We previously reported that two types of missense mutations in the highly conserved serine/threonine kinase domain of *EL1* exist in the *japonica* cultivars, H143 (type 2) and Koshihikari (type 3) (Kwon et al. 2014). Both natural mutations in *EL1* cause a loss of kinase activity, leading to a failure in phosphorylation of SLR1 (Dai and Xue 2010; Kwon et al. 2014). Consistent with the *el1* mutant phenotype, Koshihikari does show early flowering under long-day conditions (Matsubara et al. 2008; Hori et al. 2013; Kwon et al. 2014). Thus, we expected that Koshihikari would exhibit a similar defect in GA signaling and a low seed setting rate. However, Koshihikari showed no significant difference in the yield and yield-related components compared with the high-yield *japonica* cultivar ‘Dongjin (type 1; functional *EL1*)’ or the high-yield *indica* cultivar ‘Milyang23 (type 4; functional *EL1*)’ (Kwon et al. 2014; Table 1). In particular, the seed setting rate of Koshihikari was close to 90 %, suggesting no negative effect of the *el1* mutation on anther development or spikelet fertility in Koshihikari. We also found by observation of α-amylase activity that the response to GA was not altered at all in the seeds of Koshihikari compared to that of the elite *japonica* cultivar Nipponbare (Fig. 5). Moreover, the transcript levels of *GAMYB* showed no significant increase in the germinating seeds of Koshihikari, in contrast to the high *GAMYB* transcript levels in ZH11(*el1*) and HNIL(H143) (Fig. 6).

**Table 1:** Agronomic traits of three rice cultivars in Korea and Japan

Cultivar	Main panicle length (cm)	No. of panicles/plant	No. of spikelets/main panicle	500-seed weight (g)	Seed setting rate (%)	Yield/plant (g)
Dongjin (j)	20.2 ± 0.8	9.4 ± 1.7	124.6 ± 12.6	12.6 ± 0.9	93.6 ± 1.7	23.4 ± 4.3
Milyang23 (i)	25.8 ± 1.7	9.8 ± 1.7	186.7 ± 27.4	11.4 ± 0.8	84.9 ± 1.8	35.5 ± 4.7
Koshihikari (j)	19.7 ± 0.9	12.6 ± 2.5	121.9 ± 12.8	11.0 ± 1.1	88.8 ± 2.2	25.3 ± 3.2

Twenty plants were grown in Suwon, Korea (37°N) in 2014. The (j) and (i) indicate *japonica* and *indica*, respectively

## Discussion

### *EL1* Is Essential For Anther Development

Many reports have shown that GA signaling affects floral organ development, since defects in GA signaling often result in male sterility; indeed, several GA-insensitive or GA-deficient mutants exhibit anther deformities and/or produce sterile flowers in rice, petunia, maize, tomato, barley, and Arabidopsis (Lanahan and Ho 1988; Nester and Zeevaart 1988; Jacobsen and Olszewski 1991; Evans and Poethig 1995; Goto and Pharis 1999; Ikeda et al. 2001; Izhaki et al. 2002; Cheng et al. 2004; Kaneko et al. 2004; Chhun et al. 2007). Moreover, some factors in the GA signaling cascade affect flower development, particularly anther formation in rice (Kaneko et al. 2004; Chhun et al. 2007; Aya et al. 2009).

EL1/CKI has a significant role in alleviating the response to GA signaling by phosphorylating the DELLA protein SLR1, a major repressor of GA signaling in rice (Dai and Xue 2010). In ZH11(*el1*) plants, the non-phosphorylated SLR1 is degraded rapidly, suggesting that the phosphorylation of SLR1 by EL1 is essential for SLR1 stability (Dai and Xue 2010). The failure of post-translational modification of SLR1 protein triggered derepression of its downstream genes, including *GAMYB*, in HNIL(H143) (Fig. 2), leading to an increase in responses downstream of GA signaling, such as α-amylase activity in the endosperm of germinating seeds (Fig. 3 and Additional file 3: Figure S3).

The GA signaling transducer GAMYB activates expression of α-amylase in the aleurone cells of cereal grains by directly binding to the promoter of the gene encoding α-amylase (Gubler et al. 1995; Gubler et al. 1999; Gubler et al. 2002; Aya et al. 2009). GAMYB also participates in anther development and acts as a transcription factor by directly binding to the promoters of several GA-regulated genes that are responsible for exine and Ubisch body formation (Kaneko et al. 2004; Aya et al. 2009). The *gamyb* mutants show defects in floral organs, especially in anther development (Kaneko et al. 2004), because the expression of *CYP703A3* and *KAR*, which are required for normal development of anther and pollen, was dramatically suppressed in *gamyb* mutants (Aya et al. 2009). Paradoxically, overexpression of *GAMYB* in barley also causes defects in anther development, such as impaired dehiscence, decreased anther length, and lighter color (Murray et al. 2003), similar to the phenotype of *gamyb* mutants.

In this study, we showed that the transcript levels of *GAMYB* increased significantly in HNIL(H143), possibly due to a lack of SLR1 activity caused by the *el1* mutation (Fig. 2); this may cause the abnormal development of anthers observed in *el1* mutants (Fig. 1). These results suggest that normal anther development requires the precise spatiotemporal control of *GAMYB* transcription. Although further study is needed, our present results provide evidence that the alteration in the response to GA caused by the *el1* mutation impairs anther development, in addition to causing early flowering under non-inductive long-day conditions in temperate and cooler regions (Kwon et al. 2014).

### Mutation Of *EL1* Negatively Affects Spikelet Fertility

Plants that have excessive GA-induced signaling show decreased pollen viability and a sterile phenotype. For example, transgenic barley overexpressing *HvGAMYB* showed decreased spikelet fertility, and thus a severe reduction in grain yield (Murray et al. 2003). The rice *gamyb* null mutants had fewer spikelets per panicle and a defect in the induction of α-amylase expression in response to GA treatment (Kaneko et al. 2004). Similarly, the loss-of-function *slr1-1* and the gain-of-function *Slr1-d3* mutants exhibit sterile and semi-fertile phenotypes, respectively, in rice (Ikeda et al. 2001; Chhun et al. 2007). Although *Slr1-d3* mutants produced normal floral organs with morphologically normal pistils and stamens, they showed low pollen viability compared to wild type, leading to semi-fertility in the *Slr1-d3* mutants (Chhun et al. 2007).

Our further phenotypic characterization showed that many agronomic traits related to yield and yield components were lower in HNIL(H143) compared with the *EL1* HNIL(M23). Especially, the seed setting rate was significantly reduced, leading to a reduction of grain yield in HNIL(H143), suggesting that the HNIL(H143) *el1* allele affects yield and yield components (Fig. 4). The decrease of seed setting rate was also found in H143 and ZH11(*el1*), indicating that the *el1* mutation negatively affects spikelet fertility, because of low pollen viability (Additional file 1: Figure S1, Additional file 2: Figure S2, Additional file 5: Figure S5 and Additional file 6: Figure S6). Taken together, these results indicate that a loss of function in EL1/CKI decreases SLR1 activity and stability, inducing a constitutive GA response like the rice *slr1-1* mutants (Ikeda et al. 2001; Murray et al. 2003; Dai and Xue 2010). Therefore, it is considered that excessive GA signaling caused by the *el1* mutation negatively affects seed setting rate.

### No Defect Of GA Signaling In Koshihikari Carrying The *el1* (Type 3) Null Allele

**Fig. 7:**
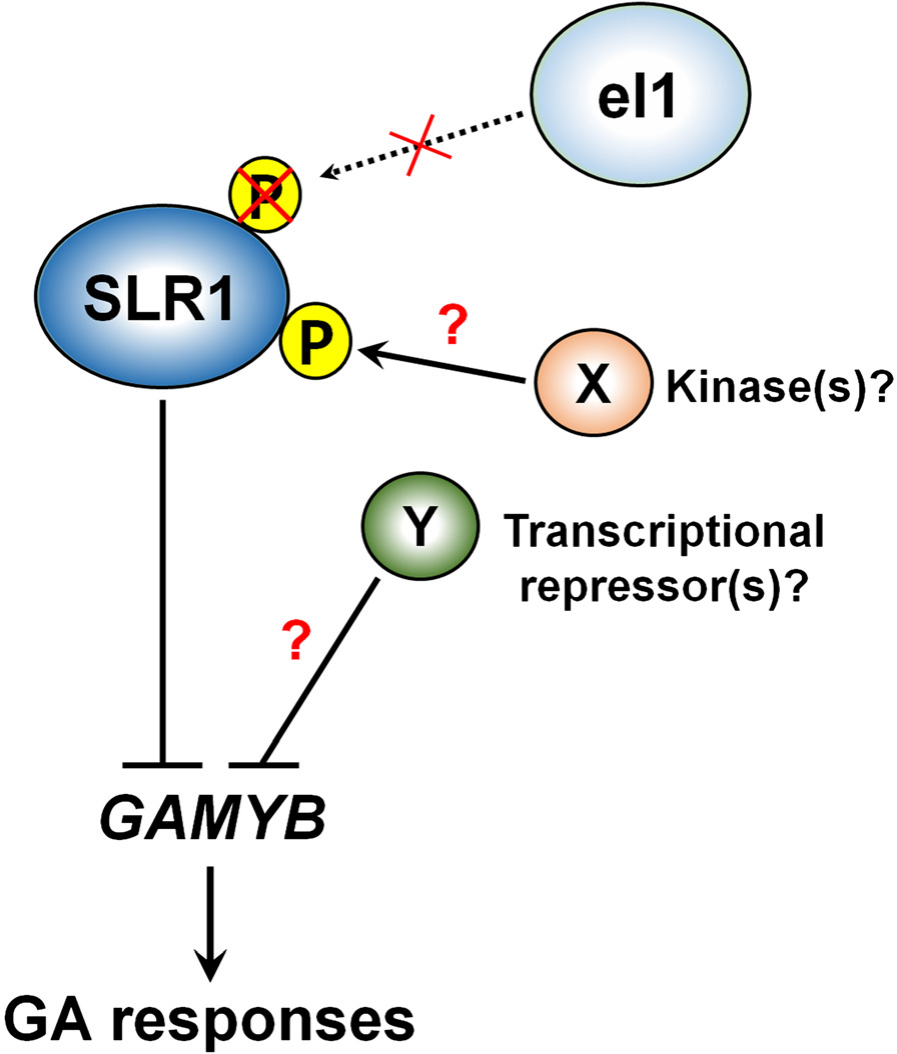
A schematic model of the GA response in Koshihikari. In the Japanese elite *japonica* cultivar ‘Koshihikari’, which carries an *el1* mutation, the phosphorylation of SLR1 by EL1 is impaired. We speculate that *GAMYB* transcription and GA responses are normally regulated by an as-yet-unidentified kinase or kinases (X) that phosphorylate SLR1 or repressor(s) of *GAMYB* expression (Y) to produce the high spikelet fertility observed in Koshihikari. P, phosphorus. Arrow and blocked lines indicate positive and negative regulation, respectively

We previously reported that naturally occurring *el1* alleles (types 2 and 3) contribute to rice adaptation to growth in the northernmost, cooler regions by inducing photoperiod insensitivity and early flowering under long-day conditions (Kwon et al. 2014). In addition to the positive effect of *el1* in rice domestication and adaptation, this study also reports its negative effect on spikelet fertility. However, by investigating the response to GA signaling and yield-related traits of Koshihikari plants, which carry a nonfunctional *el1* (type 3) allele (Hori et al. 2013; Kwon et al. 2014), we found that the Koshihikari cultivar shows a normal GA response and seed setting rate, compared with other elite *japonica* cultivars possessing a functional *EL1* allele (Fig. 5 and Table 1). In addition, *GAMYB* expression in Koshihikari was almost the same as that of Nipponbare, ZH11, and HNIL(M23), in contrast with the higher *GAMYB* expression observed in ZH11(*el1*) and HNIL(H143) (Fig. 6). This finding strongly suggests that *GAMYB* expression and/or GA signaling is controlled normally in Koshihikari, although the *el1* (type 3) mutation contributes to the early flowering of Koshihikari under long-day conditions in temperate regions. In Arabidopsis, mutants for all five DELLA paralogs still show basal levels of fertility in the Landsberg *erecta* (L*er*) ecotype, indicating that DELLA activity in L*er* is not essential to maintain fertility (Dill and Sun 2001; Fuentes et al. 2012). In contrast, the *rga gai* mutants in the Columbia (Col-0) ecotype show male sterility induced by post-meiotic defects in pollen development, and the sterile phenotype are recovered by transgenic introduction of DELLAs into developing pollen (Plackett et al. 2014). Furthermore, the distinct fertility phenotype of Col-0 and L*er* can be caused by either differences in downstream signaling mechanisms or altered expression of DELLA proteins (Plackett et al. 2014). Thus, we propose that the introduction or natural variation of other unknown regulator(s) offsetting excessive GA signaling may have occurred during the breeding of Koshihikari. These unknown regulators act downstream of *SLR1* in GA signaling, or directly in SLR1 protein modification and stability (Fig. 7). Therefore, further studies to identify the unknown factor(s) are ongoing to explain how Koshihikari can achieve normal GA signaling and thus maintain spikelet fertility in the *el1* mutant background.

### Pleiotropy Of The *el1* Mutation In Rice Development

Two recent reports showed the molecular mechanism of *EL1* function in the photoperiodic pathway in rice flowering (Hori et al. 2013; Kwon et al. 2014). In addition, it had been published that EL1 functions in GA signaling by demonstrating the in vitro phosphorylation of SLR1 (Dai and Xue 2010). EL1/CKI acts as a flowering repressor by interacting with and directly phosphorylating Ghd7 and OsPRR37; this modification delays heading date in non-inductive long-day conditions (Hori et al. 2013; Kwon et al. 2015). This suggests that the targets downstream of EL1/CKI act throughout various genetic and signaling pathways during rice development, such as inflorescence formation, flowering, stem elongation, and germination. Moreover, the *el1* mutant displays other phenotypes related to GA action, such as taller plants and longer epidermal cells (Dai and Xue 2010). In this study, we demonstrate that the *el1* mutation decreases grain yield because of a reduction of seed setting rate.

## Conclusion

Although the *el1* mutation contributes to early flowering of *japonica* rice cultivars under non-inductive long-day conditions in temperate and cooler regions, it can also negatively affect grain production. The enhanced response to GA signaling in the rice *el1* mutants induces overexpression of *GAMYB* and the pollen formation-related genes *CYP703A3* and *KAR* during spikelet development, causing defects in anther development and pollen viability. However, the elite *japonica* cultivar Koshihikari, harboring an *el1* allele, appears to overcome this disadvantage by having unknown regulator(s) that maintain normal GA signaling. This may explain why the *el1* allele occurs in only a few extremely early-flowering *japonica* cultivars in the world rice collections (Kwon et al. 2014).

## Methods

### Plant Materials And Growth Conditions

Heterogeneous inbred family-near isogenic lines (HNILs) derived from crosses between a *japonica* rice ‘H143’ and a *japonica*-*indica* hybrid rice ‘M23’ were used in this study (Koo et al. 2013; Kwon et al. 2014). The *EL1* and *el1* alleles of HNIL(M23) and HNIL(H143) were derived from M23 and H143, respectively (Kwon et al. 2014). H143, M23, ZH11, and ZH11(*el1*) (Dai and Xue 2010) were grown for observation of spikelet structure and analysis of agronomic traits. The plants for this study were grown in the paddy field of Seoul National University experimental farm in Suwon, Korea (37°N latitude) from April to October. The day lengths of Suwon were about 13.5 h (April), 14.2 h (May), 14.7 h (June), 14.3 h (July) and 13.6 h (August). Cultivation of these plants followed standard agricultural methods.

### Reverse Transcription And Quantitative Real-Time PCR (RT-qPCR)

Total RNA from spikelets or germinating seeds was extracted using the Total RNA Extraction Kit (Macrogen, Korea). First-strand cDNA was synthesized from 2 μg total RNA using oligo(dT)_15_ primer and M-MLV reverse transcriptase (Promega, Madison, WI, USA). Transcript levels of GA signaling-related genes were detected by qPCR using gene-specific primers and *Ubiquitin5* (*UBQ5*) as the internal control (Additional file 7: Table S1). Reactions of 20 μl included 2 μl of 0.5 μM primer, 2 μl of cDNA mixture, and 10 μl of 2X QuantiTect LightCycler 480 SYBR Green I Master Mix (Roche, Basel, Switzerland). qPCR was performed with the Light Cycler 2.0 instrument (Roche) using the program: 95 °C for 2 min, 45 cycles of 95 °C for 10 s, 59 °C for 10 s, and 72 °C for 10 s.

### α-Amylase Assay

The α-amylase assay was performed as previously described (Yamaguchi 1998; Dai and Xue 2010). Briefly, seed coats and embryos were removed and only endosperms were sterilized in 1 % NaClO containing 0.01 % TWEEN 20 for 30 min, and then rinsed with distilled water 6 times. The half-seeds were placed on 2 % phytoagar medium containing 0.2 % soluble starch, and media including GA_3_ or ABA were used for analysis. All the plates were incubated in the dark at 28 °C for 3 days. After that, the endosperms were removed from the plates, and then the plates were stained with 0.1 % iodine and 1 % potassium iodide. Seeds with α-amylase activity were quantified by counting white spots.

### Pollen Viability Analysis

To determine pollen viability, six anthers were picked from a spikelet at heading stage and moved to a glass slide. The anthers were broken and stained with 0.1 % iodine and 1 % potassium iodide solution (I_2_-KI). Five minutes later, the pollen grains were observed using an optical microscope (Olympus, BX50). Viable pollen grains were identified by their black color and round shape; sterile pollen grains were identified by their yellow color and shrunken shape.

## Additional Files

Additional file 1: Figure S1. Flower and pollen structure of ZH11 and ZH11(*el1*) plants. (**A**) Panicle structure of ZH11 and ZH11(*el1*) at the heading stage. Scale bar = 20 mm. (**B**) Spikelets of ZH11 and ZH11(*el1*). Scale bar = 20 mm. (**C**) Flowers of the ZH11 and ZH11(*el1*) plants. An, anther; Le, lemma; Fl, filament; Pl, palea. Scale bar = 20 mm. (**D**) Anthers of the ZH11 and ZH11(*el1*) plants. Scale bar = 10 mm. (**E**) Pistils of the ZH11 and ZH11(*el1*) plants. Scale bar = 1 mm. The pollen grains from (**F**) ZH11 and (**G**) ZH11(*el1*) plants. Pollen was stained with I_2_-KI solution. Scale bar = 200 μm (**F** and **G**). The data represent five independent biological replicates. (DOCX 1785 kb) Additional file 2: Figure S2. Flower and pollen structure of H143 and M23. (**A**) Panicle structure of M23 and the H143 at the heading stage. Scale bar = 20 mm. (**B**) Spikelets of the M23 and H143 plants. Scale bar = 20 mm. (**C**) Flowers of the M23 and H143 plants. An, anther; Le, lemma; Fl, filament; Pl, palea. Scale bar = 20 mm. (**D**) Anthers of the M23 and H143 plants. Scale bar = 10 mm. (**E**) Pistils of the M23 and H143 plants. Scale bar = 1 mm. Pollen grains from (**F**) M23 and (**G**) H143 plants. Pollen stained with I_2_-KI solution. Scale bar = 200 μm (**F** and **G**). The data represent five independent biological replicates. (DOCX 1774 kb) Additional file 3: Figure S3. Assay for α-amylase activity on starch plates. Production of α-amylase from the endosperm of seeds was detected as white spots. The starch plates also contained 10 μM ABA or 1 mM GA_3_. The plates were stained with 0.1 % iodine and 1 % potassium iodide solution. (**A**) HNIL(M23) and HNIL(H143) seeds; (**B**), M23 and H143 seeds. These experiments were repeated at least three times. (DOCX 1006 kb) Additional file 4: Figure S4. Yield-related agronomic traits of HNIL(M23) and HNIL(H143) (F_7:11_). Agronomic traits were measured in HNIL(M23) and HNIL(H143) plants grown under natural long days in 2012. The measured traits were: (**A**) main panicle length, (**B**) number of panicles per plant, (**C**) number of spikelets per main panicle, (**D**) 500-grain weight, (**E**) yield per plant, and (**F**) seed setting rate. 20 plants were used to measure each trait. Student’s *t*-test was used for statistical analysis (**P* < 0.05, ***P* < 0.01, ****P* < 0.001). Means and standard deviations are marked as values and vertical bars, respectively. (**G**) Fertile and sterile seeds from the whole plants of HNIL(M23) and HNIL(H143). Scale bar = 2 cm. (DOCX 1896 kb) Additional file 5: Figure S5. Yield-related agronomic traits of M23 and H143. Agronomic traits were measured in M23 and H143 plants grown under natural long days in 2013. The measured traits were: (**A**) main panicle length, (**B**) number of panicles per plant, (**C**) number of spikelets per main panicle, (**D**) 500-grain weight, (**E**) yield per plant and (F) seed setting rate. 20 plants were used to measure each trait. Student’s *t*-test was used for statistical analysis (**P* < 0.05, ***P* < 0.01, ****P* < 0.001). Means and standard deviations are marked as values and vertical bars, respectively. (**G**) Fertile and sterile seeds from the whole plants of M23 and H143. Scale bar = 2 cm. (DOCX 1639 kb) Additional file 6: Figure S6. Yield-related agronomic traits of ZH11 and ZH11(*el1*). Agronomic traits were measured in ZH11 and ZH11(*el1*) grown under natural long days in 2014. The measured traits were: (**A**) main panicle length, (**B**) number of panicles per plant, (**C**) number of spikelets per main panicle, (**D**) 500-grain weight, (**E**) yield per plant and (**F**) seed setting rate. 10 plants were used to measure each trait. Student’s *t*-test was used for statistical analysis (**P* < 0.05, ** *P* < 0.01). Means and standard deviations are marked as values and vertical bars, respectively. (**G**) Fertile and sterile seeds from the whole plants of ZH11 and ZH11(*el1*). Scale bar = 2 cm. (DOCX 1723 kb) Additional file 7: Table S1. Primers for RT-qPCR. (DOCX 22 kb)

## Abbreviations

CKI: Casein kinase I
CYP703A3: Cytochrome P450 hydroxylase
EL1: Early flowering1
HNIL: Heterogeneous inbred family-near isogenic line
KAR: β-ketoacyl reductase
M23: Milyang23
OsPRR37: Oryza sativa pseudo-response regulator37
SLR1: Slender rice1

## References

[CR1] Achard P, Herr A, Baulcombe DC, Harberd NP (2004). Modulation of floral development by a gibberellin-regulated microRNA. Development.

[CR2] Aya K, Ueguchi-Tanaka M, Kondo M, Hamada K, Yano K, Nishimura M, Matsuoka M (2009). Gibberellin modulates anther development in rice via the transcriptional regulation of GAMYB. Plant Cell.

[CR3] Aya K, Suzuki G, Suwabe K, Hobo T, Takahashi H, Shiono K, Yano K, Tsutsumi N, Nakazono M, Nagamura Y, Matsuoka M, Watanabe M (2011). Comprehensive network analysis of anther-expressed genes in rice by the combination of 33 laser microdissection and 143 spatiotemporal microarrays. PLoS One.

[CR4] Cecchetti V, Altamura MM, Falasca G, Costantino P, Cardarelli M (2008). Auxin regulates *Arabidopsis* anther dehiscence, pollen maturation, and filament elongation. Plant Cell.

[CR5] Chen CB, Xu YY, Ma H, Chong K (2005). Cell biological characterization of male meiosis and pollen development in rice. J Integr Plant Biol.

[CR6] Cheng H, Qin LJ, Lee SC, Fu XD, Richards DE, Cao DN, Luo D, Harberd NP, Peng JR (2004). Gibberellin regulates *Arabidopsis* floral development via suppression of DELLA protein function. Development.

[CR7] Chhun T, Aya K, Asano K, Yamamoto E, Morinaka Y, Watanabe M, Kitano H, Ashikari M, Matsuoka M, Ueguchi-Tanaka M (2007). Gibberellin regulates pollen viability and pollen tube growth in rice. Plant Cell.

[CR8] Dai C, Xue HW (2010). Rice *early flowering*1, a CKI, phosphorylates DELLA protein SLR1 to negatively regulate gibberellin signalling. EMBO J.

[CR9] Dill A, Sun TP (2001). Synergistic derepression of gibberellin signaling by removing RGA and GAI function in *Arabidopsis thaliana*. Genetics.

[CR10] Evans MM, Poethig RS (1995). Gibberellins promote vegetative phase change and reproductive maturity in maize. Plant Physiol.

[CR11] Fleet CM, Sun TP (2005). A DELLAcate balance: the role of gibberellin in plant morphogenesis. Curr Opin Plant Biol.

[CR12] Fuentes S, Ljung K, Sorefan K, Alvey E, Harberd NP, Ostergaard L (2012). Fruit growth in *Arabidopsis* occurs via DELLA-dependent and DELLA-independent gibberellin responses. Plant Cell.

[CR13] Gomi K, Sasaki A, Itoh H, Ueguchi-Tanaka M, Ashikari M, Kitano H, Matsuoka M (2004). GID2, an F-box subunit of the SCF E3 complex, specifically interacts with phosphorylated SLR1 protein and regulates the gibberellin-dependent degradation of SLR1 in rice. Plant J.

[CR14] Goto N, Pharis RP (1999). Role of gibberellins in the development of floral organs of the gibberellin-deficient mutant, *ga1-1*, of *Arabidopsis thaliana*. Can J Bot.

[CR15] Gubler F, Kalla R, Roberts JK, Jacobsen JV (1995). Gibberellin-regulated expression of a *myb* gene in barley aleurone cells: Evidence for Myb transactivation of a high-pl α-amylase gene promoter. Plant Cell.

[CR16] Gubler F, Raventos N, Keys M, Watts R, Mundy J, Jacobsen JV (1999). Target genes and regulatory domains of the GAMYB transcriptional activator in cereal aleurone. Plant J.

[CR17] Gubler F, Chandler PM, White RG, Llewellyn DJ, Jacobsen JV (2002). Gibberellin signaling in barley aleurone cells. Control of SLN1 and GAMYB expression. Plant Physiol.

[CR18] Guo JX, Liu YG (2012). Molecular control of male reproductive development and pollen fertility in rice. J Integr Plant Biol.

[CR19] Hedden P, Phillips AL (2000). Gibberellin metabolism: new insights revealed by the genes. Trends Plant Sci.

[CR20] Hirano K, Aya K, Hobo T, Sakakibara H, Kojima M, Shim RA, Hasegawa Y, Ueguchi-Tanaka M, Matsuoka M (2008). Comprehensive transcriptome analysis of phytohormone biosynthesis and signaling genes in microspore/pollen and tapetum of rice. Plant Cell Physiol.

[CR21] Hori K, Ogiso-Tanaka E, Matsubara K, Yamanouchi U, Ebana K, Yano M (2013). Hd16, a gene for casein kinase I, is involved in the control of rice flowering time by modulating the day-length response. Plant J.

[CR22] Hu JH, Mitchum MG, Barnaby N, Ayele BT, Ogawa M, Nam E, Lai WC, Hanada A, Alonso JM, Ecker JR, Swain SM, Yamaguchi S, Kamiya Y, Sun TP (2008). Potential sites of bioactive gibberellin production during reproductive growth in *Arabidopsis*. Plant Cell.

[CR23] Ikeda A, Ueguchi-Tanaka M, Sonoda Y, Kitano H, Koshioka M, Futsuhara Y, Matsuoka M, Yamaguchi J (2001). Slender rice, a constitutive gibberellin response mutant, is caused by a null mutation of the SLR1 gene, an ortholog of the height-regulating gene *GAI*/*RGA*/*RHT*/*D8*. Plant Cell.

[CR24] Itoh H, Ueguchi-Tanaka M, Sato Y, Ashikari M, Matsuoka M (2002). The gibberellin signaling pathway is regulated by the appearance and disappearance of SLENDER RICE1 in nuclei. Plant Cell.

[CR25] Itoh H, Sasaki A, Ueguchi-Tanaka M, Ishiyama K, Kobayashi M, Hasegawa Y, Minami E, Ashikari M, Matsuoka M (2005). Dissection of the phosphorylation of rice DELLA protein, SLENDER RICE1. Plant Cell Physiol.

[CR26] Itoh J, Nonomura K, Ikeda K, Yamaki S, Inukai Y, Yamagishi H, Kitano H, Nagato Y (2005). Rice plant development: from zygote to spikelet. Plant Cell Physiol.

[CR27] Izhaki A, Borochov A, Zamski E, Weiss D (2002). Gibberellin regulates post-microsporogenesis processes in petunia anthers. Physiol Plant.

[CR28] Jacobsen SE, Olszewski NE (1991). Characterization of the arrest in anther development associated with gibberellin deficiency of the *gib-1* mutant of tomato. Plant Physiol.

[CR29] Kaneko M, Inukai Y, Ueguchi-Tanaka M, Itoh H, Izawa T, Kobayashi Y, Hattori T, Miyao A, Hirochika H, Ashikari M, Matsuoka M (2004). Loss-of-function mutations of the rice *GAMYB* gene impair α-amylase expression in aleurone and flower development. Plant Cell.

[CR30] Koo BH, Yoo SC, Park JW, Kwon CT, Lee BD, An G, Zhang ZY, Li JJ, Li ZC, Paek NC (2013). Natural variation in *OsPRR37* regulates heading date and contributes to rice cultivation at a wide range of latitudes. Mol Plant.

[CR31] Kwon CT, Yoo SC, Koo BH, Cho SH, Park JW, Zhang ZY, Li JJ, Li ZC, Paek NC (2014). Natural variation in *Early flowering1* contributes to early flowering in japonica rice under long days. Plant Cell Environ.

[CR32] Kwon CT, Koo BH, Kim D, Yoo SC, Paek NC (2015). Casein kinases I and 2α phosphorylate Oryza sativa pseudo-response regulator 37 (OsPRR37) in photoperiodic flowering in rice. Mol Cells.

[CR33] Lanahan MB, Ho THD (1988). Slender barley - a constitutive gibberellin-response mutant. Planta.

[CR34] Liu W, Zhang DC, Tang MF, Li DY, Zhu YX, Zhu LH, Chen CY (2013). THIS1 is a putative lipase that regulates tillering, plant height, and spikelet fertility in rice. J Exp Bot.

[CR35] Ma H (2005). Molecular genetic analyses of microsporogenesis and microgametogenesis in flowering plants. Annu Rev Plant Biol.

[CR36] Matsubara K, Kono I, Hori K, Nonoue Y, Ono N, Shomura A, Mizubayashi T, Yamamoto S, Yamanouchi U, Shirasawa K, Nishio T, Yano M (2008). Novel QTLs for photoperiodic flowering revealed by using reciprocal backcross inbred lines from crosses between japonica rice cultivars. Theor Appl Genet.

[CR37] McCormick S (2004). Control of male gametophyte development. Plant Cell.

[CR38] Millar AA, Gubler F (2005). The Arabidopsis *GAMYB-like* genes, *MYB33* and *MYB65*, are microRNA-regulated genes that redundantly facilitate anther development. Plant Cell.

[CR39] Murray F, Kalla R, Jacobsen J, Gubler F (2003). A role for HvGAMYB in anther development. Plant J.

[CR40] Nester JE, Zeevaart JAD (1988). Flower development in normal tomato and a gibberellin-deficient (*ga-2*) mutant. Am J Bot.

[CR41] Park JH, Halitschke R, Kim HB, Baldwin IT, Feldmann KA, Feyereisen R (2002). A knock-out mutation in allene oxide synthase results in male sterility and defective wound signal transduction in *Arabidopsis* due to a block in jasmonic acid biosynthesis. Plant J.

[CR42] Plackett ARG, Thomas SG, Wilson ZA, Hedden P (2011). Gibberellin control of stamen development: a fertile field. Trends Plant Sci.

[CR43] Plackett ARG, Ferguson AC, Powers SJ, Wanchoo-Kohli A, Phillips AL, Wilson ZA, Hedden P, Thomas SG (2014). DELLA activity is required for successful pollen development in the Columbia ecotype of Arabidopsis. New Phytol.

[CR44] Ritchie S, Gilroy S (1998). Gibberellins: regulating genes and germination. New Phytol.

[CR45] Sasaki A, Itoh H, Gomi K, Ueguchi-Tanaka M, Ishiyama K, Kobayashi M, Jeong DH, An G, Kitano H, Ashikari M, Matsuoka M (2003). Accumulation of phosphorylated repressor for gibberellin signaling in an F-box mutant. Science.

[CR46] Scott RJ, Spielman M, Dickinson HG (2004). Stamen structure and function. Plant Cell.

[CR47] Shimada A, Ueguchi-Tanaka M, Sakamoto T, Fujioka S, Takatsuto S, Yoshida S, Sazuka T, Ashikari M, Matsuoka M (2006). The rice *SPINDLY* gene functions as a negative regulator of gibberellin signaling by controlling the suppressive function of the DELLA protein, SLR1, and modulating brassinosteroid synthesis. Plant J.

[CR48] Singh DP, Jermakow AM, Swain SM (2002). Gibberellins are required for seed development and pollen tube growth in Arabidopsis. Plant Cell.

[CR49] Thangasamy S, Guo CL, Chuang MH, Lai MH, Chen JC, Jauh GY (2011). Rice *SIZ1*, a SUMO E3 ligase, controls spikelet fertility through regulation of anther dehiscence. New Phytol.

[CR50] Tsuji H, Aya K, Ueguchi-Tanaka M, Shimada Y, Nakazono M, Watanabe R, Nishizawa NK, Gomi K, Shimada A, Kitano H, Ashikari M, Matsuoka M (2006). GAMYB controls different sets of genes and is differentially regulated by microRNA in aleurone cells and anthers. Plant J.

[CR51] Ueguchi-Tanaka M, Ashikari M, Nakajima M, Itoh H, Katoh E, Kobayashi M, Chow TY, Hsing YIC, Kitano H, Yamaguchi I, Matsuoka M (2005). *GIBBERELLIN INSENSITIVE DWARF1* encodes a soluble receptor for gibberellin. Nature.

[CR52] Ueguchi-Tanaka M, Nakajima M, Katoh E, Ohmiya H, Asano K, Saji S, Xiang HY, Ashikari M, Kitano H, Yamaguchi I, Matsuokaa M (2007). Molecular interactions of a soluble gibberellin receptor, GID1, with a rice DELLA protein, SLR1, and gibberellin. Plant Cell.

[CR53] Ueguchi-Tanaka M, Nakajima M, Motoyuki A, Matsuoka M (2007). Gibberellin receptor and its role in gibberellin signaling in plants. Annu Rev Plant Biol.

[CR54] Wilson ZA, Zhang DB (2009). From *Arabidopsis* to rice: pathways in pollen development. J Exp Bot.

[CR55] Woodger FJ, Millar A, Murray F, Jacobsen JV, Gubler F (2003). The role of GAMYB transcription factors in GA-regulated gene expression. J Plant Growth Regul.

[CR56] Yamaguchi J (1998). Analysis of embryo-specific alpha-amylase using isolated mature rice embryos. Breed Sci.

[CR57] Zhu QH, Ramm K, Shivakkumar R, Dennis ES, Upadhyaya NM (2004). The *ANTHER INDEHISCENCE1* gene encoding a single MYB domain protein is involved in anther development in rice. Plant Physiol.

