# Supplementary material for: The Rice Floral Repressor Early flowering1 Affects Spikelet Fertility By Modulating Gibberellin Signaling
Source: Rice (N Y). 2015 Jul 24;8:23. doi: 10.1186/s12284-015-0058-1 (PMC4584262; doi:10.1186/s12284-015-0058-1)
Supplement: Additional file 1: Figure S1. — Flower and pollen structure of ZH11 and ZH11(el1) plants. (A) Panicle structure of ZH11 and ZH11(el1) at the heading stage. Scale bar = 20 mm. (B) Spikelets of ZH11 and ZH11(el1). Scale bar = 20 mm. (C) Flowers of the ZH11 and ZH11(el1) plants. An, anther; Le, lemma; Fl, filament; Pl, palea. Scale bar = 20 mm. (D) Anthers of the ZH11 and ZH11(el1) plants. Scale bar = 10 mm. (E) Pistils of the ZH11 and ZH11(el1) plants. Scale bar = 1 mm. The pollen grains from (F) ZH11 and (G) ZH11(el1) plants. Pollen was stained with I2-KI solution. Scale bar = 200 μm (F and G). The data represent five independent biological replicates. (DOCX 1785 kb) [file 12284_2015_58_MOESM1_ESM.docx]

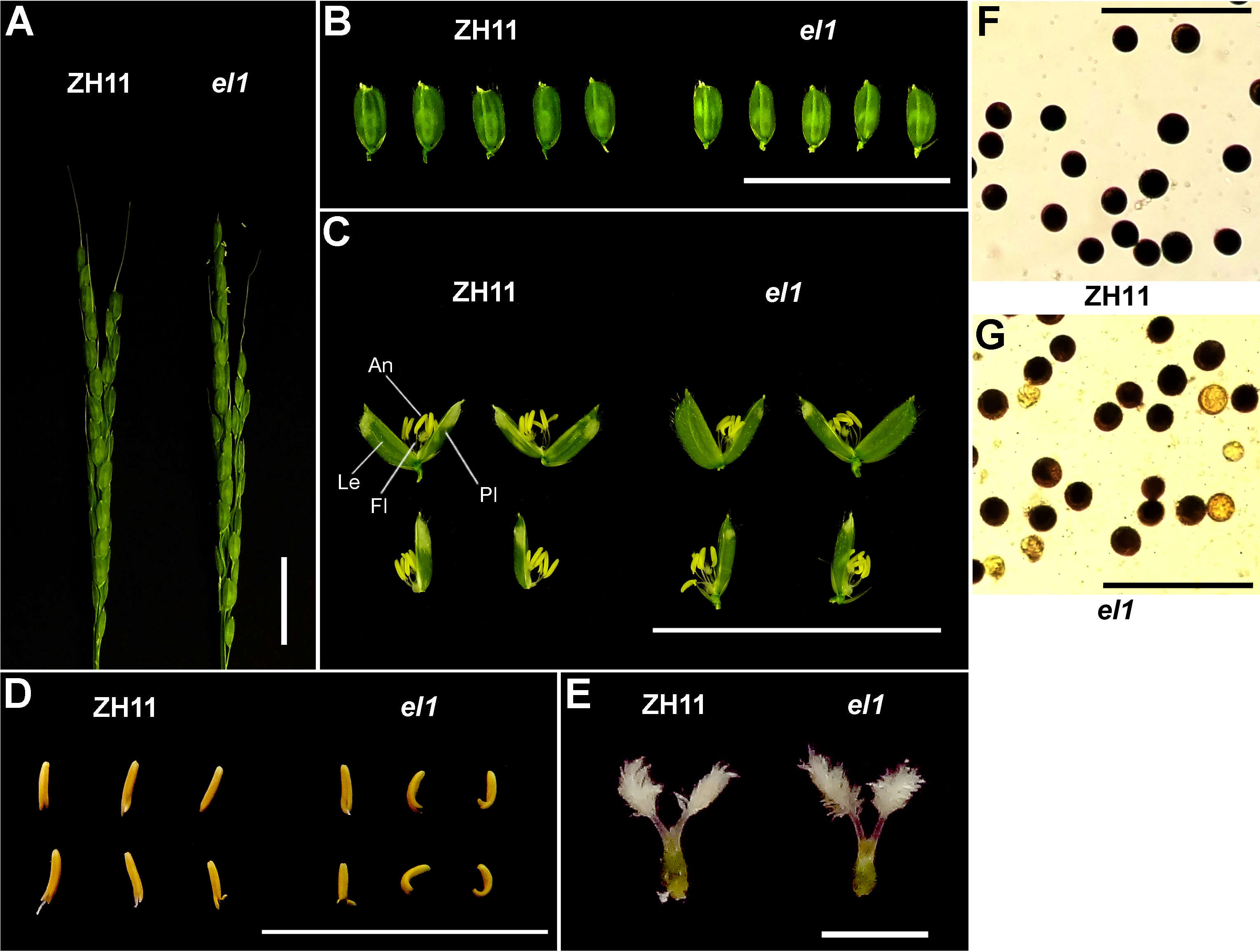


**Additional file 1: Figure S1** **Flower and pollen structure of ZH11 and ZH11(*el1*) plants.** (**A**) Panicle structure of ZH11 and ZH11(*el1*) at the heading stage. Scale bar = 20 mm. **(B)** Spikelets of ZH11 and ZH11(*el1*). Scale bar = 20 mm. **(C)** Flowers of the ZH11 and ZH11(*el1*) plants. An, anther; Le, lemma; Fl, filament; Pl, palea. Scale bar = 20 mm. **(D)** Anthers of the ZH11 and ZH11(*el1*) plants. Scale bar = 10 mm. **(E)** Pistils of the ZH11 and ZH11(*el1*) plants. Scale bar = 1 mm. The pollen grains from **(F)** ZH11 and **(G)** ZH11(*el1*) plants. Pollen was stained with I_2_-KI solution. Scale bar = 200 μm **(F and G)**. The data represent five independent biological replicates.
