# Supplementary material for: The Rice Floral Repressor Early flowering1 Affects Spikelet Fertility By Modulating Gibberellin Signaling
Source: Rice (N Y). 2015 Jul 24;8:23. doi: 10.1186/s12284-015-0058-1 (PMC4584262; doi:10.1186/s12284-015-0058-1)
Supplement: Additional file 2: Figure S2. — Flower and pollen structure of H143 and M23. (A) Panicle structure of M23 and the H143 at the heading stage. Scale bar = 20 mm. (B) Spikelets of the M23 and H143 plants. Scale bar = 20 mm. (C) Flowers of the M23 and H143 plants. An, anther; Le, lemma; Fl, filament; Pl, palea. Scale bar = 20 mm. (D) Anthers of the M23 and H143 plants. Scale bar = 10 mm. (E) Pistils of the M23 and H143 plants. Scale bar = 1 mm. Pollen grains from (F) M23 and (G) H143 plants. Pollen stained with I2-KI solution. Scale bar = 200 μm (F and G). The data represent five independent biological replicates. (DOCX 1774 kb) [file 12284_2015_58_MOESM2_ESM.docx]

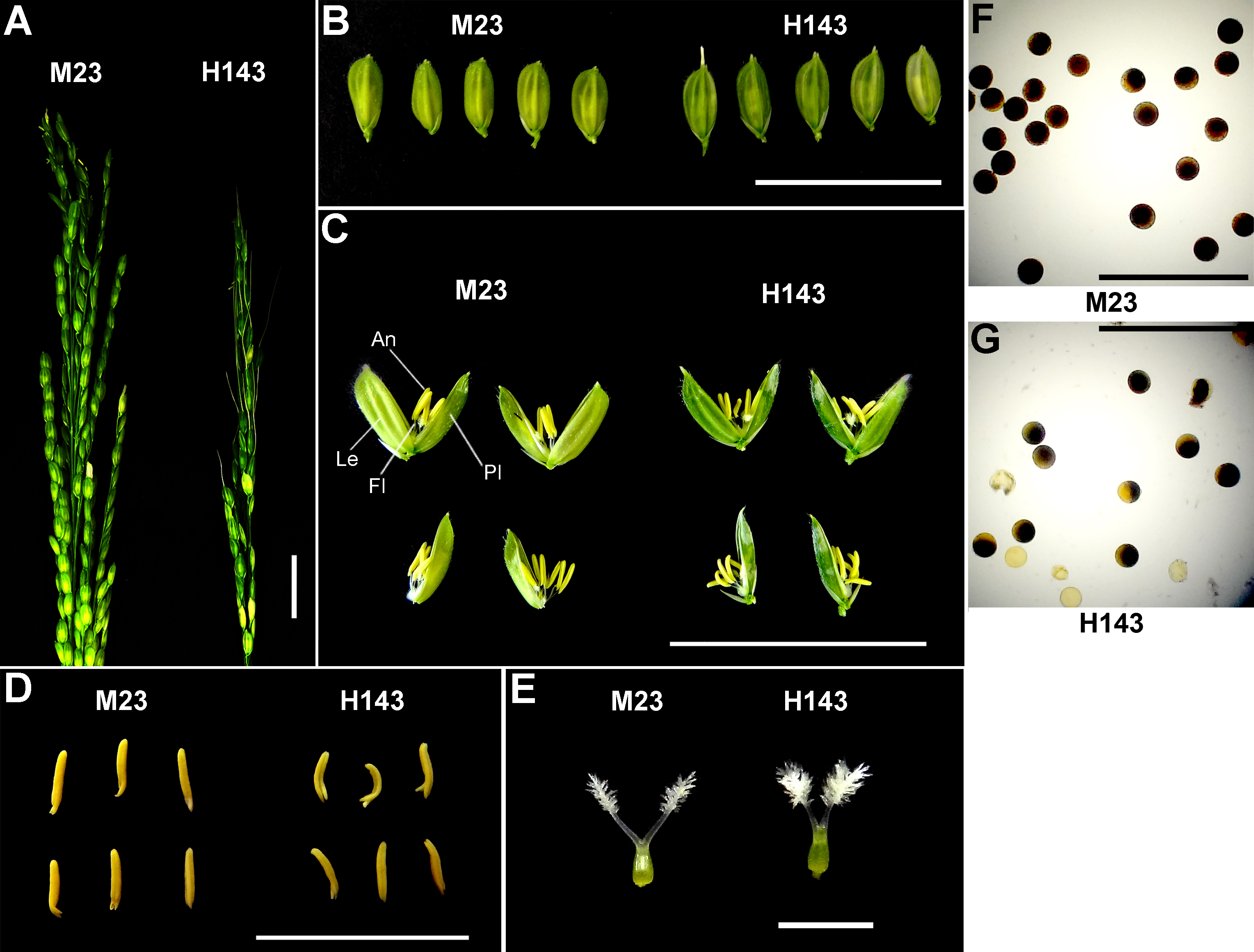


**Additional file 2: Figure S2 Flower and pollen structure of H143 and M23. (A)** Panicle structure of M23 and the H143 at the heading stage. Scale bar = 20 mm. **(B)** Spikelets of the M23 and H143 plants. Scale bar = 20 mm. **(C)** Flowers of the M23 and H143 plants. An, anther; Le, lemma; Fl, filament; Pl, palea. Scale bar = 20 mm. **(D)** Anthers of the M23 and H143 plants. Scale bar = 10 mm. **(E)** Pistils of the M23 and H143 plants. Scale bar = 1 mm. Pollen grains from **(F)** M23 and **(G)** H143 plants. Pollen stained with I_2_-KI solution. Scale bar = 200 μm **(F and G)**. The data represent five independent biological replicates.
