# Supplementary material for: The Rice Floral Repressor Early flowering1 Affects Spikelet Fertility By Modulating Gibberellin Signaling
Source: Rice (N Y). 2015 Jul 24;8:23. doi: 10.1186/s12284-015-0058-1 (PMC4584262; doi:10.1186/s12284-015-0058-1)
Supplement: Additional file 3: Figure S3. — Assay for α-amylase activity on starch plates. Production of α-amylase from the endosperm of seeds was detected as white spots. The starch plates also contained 10 μM ABA or 1 mM GA3. The plates were stained with 0.1 % iodine and 1 % potassium iodide solution. (A) HNIL(M23) and HNIL(H143) seeds; (B), M23 and H143 seeds. These experiments were repeated at least three times. (DOCX 1006 kb) [file 12284_2015_58_MOESM3_ESM.docx]

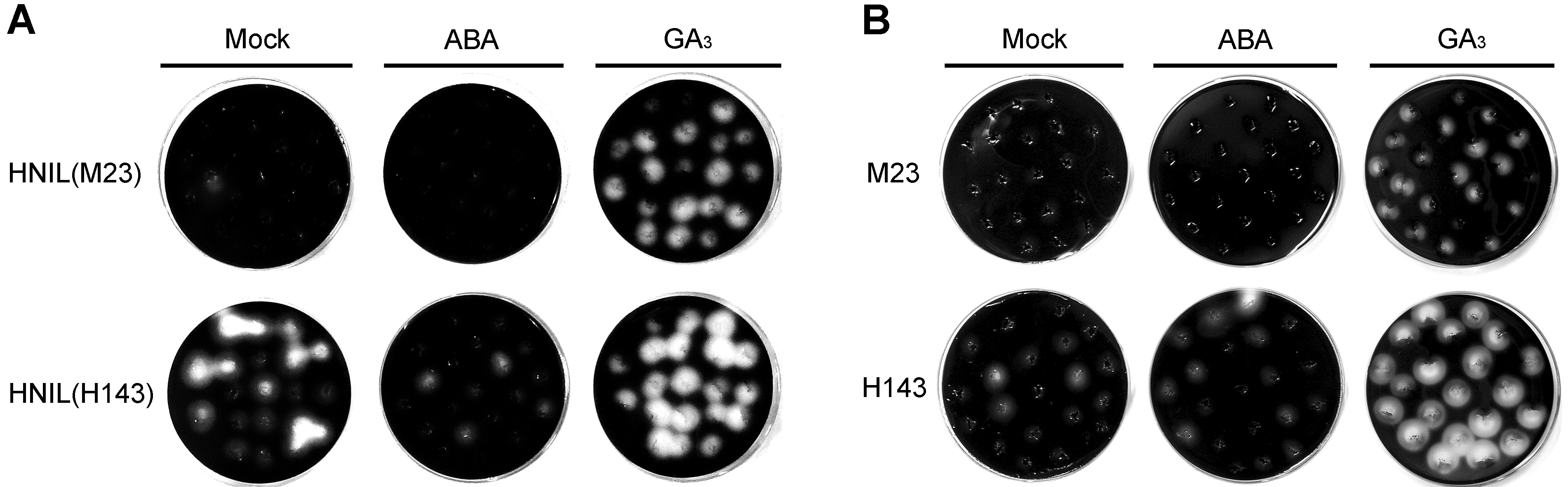


**Additional file 3: Figure S3 Assay for α-amylase activity on starch plates.** Production of α-amylase from the endosperm of seeds was detected as white spots. The starch plates also contained 10 µM ABA or 1 mM GA_3_. The plates were stained with 0.1% iodine and 1% potassium iodide solution. **(A)** HNIL(M23) and HNIL(H143) seeds; **(B)**, M23 and H143 seeds. These experiments were repeated at least three times.
