# Supplementary material for: The Rice Floral Repressor Early flowering1 Affects Spikelet Fertility By Modulating Gibberellin Signaling
Source: Rice (N Y). 2015 Jul 24;8:23. doi: 10.1186/s12284-015-0058-1 (PMC4584262; doi:10.1186/s12284-015-0058-1)
Supplement: Additional file 4: Figure S4. — Yield-related agronomic traits of HNIL(M23) and HNIL(H143) (F7:11). Agronomic traits were measured in HNIL(M23) and HNIL(H143) plants grown under natural long days in 2012. The measured traits were: (A) main panicle length, (B) number of panicles per plant, (C) number of spikelets per main panicle, (D) 500-grain weight, (E) yield per plant, and (F) seed setting rate. 20 plants were used to measure each trait. Student’s t-test was used for statistical analysis (*P < 0.05, **P < 0.01, ***P < 0.001). Means and standard deviations are marked as values and vertical bars, respectively. (G) Fertile and sterile seeds from the whole plants of HNIL(M23) and HNIL(H143). Scale bar = 2 cm. (DOCX 1896 kb) [file 12284_2015_58_MOESM4_ESM.docx]

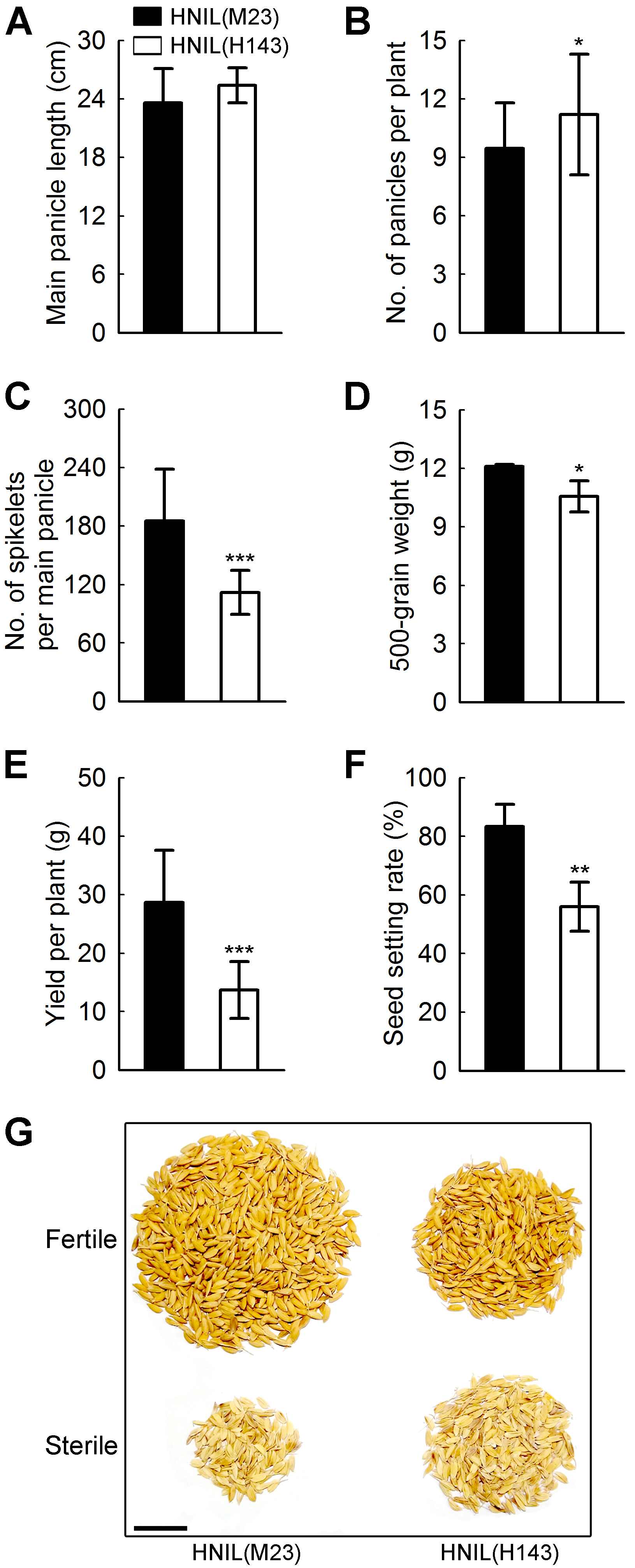


**Additional file 4: Figure S4 Yield-related agronomic traits of HNIL(M23) and HNIL(H143) (F_7:11_).** Agronomic traits were measured in HNIL(M23) and HNIL(H143) plants grown under natural long days in 2012. The measured traits were: **(A)** main panicle length, **(B)** number of panicles per plant, **(C)** number of spikelets per main panicle, **(D)** 500-grain weight, **(E)** yield per plant, and **(F)** seed setting rate. 20 plants were used to measure each trait. Student's *t*-test was used for statistical analysis (**P* < 0.05, ***P* < 0.01, ****P* < 0.001). Means and standard deviations are marked as values and vertical bars, respectively. **(G)** Fertile and sterile seeds from the whole plants of HNIL(M23) and HNIL(H143). Scale bar = 2 cm.
