# Supplementary material for: The Rice Floral Repressor Early flowering1 Affects Spikelet Fertility By Modulating Gibberellin Signaling
Source: Rice (N Y). 2015 Jul 24;8:23. doi: 10.1186/s12284-015-0058-1 (PMC4584262; doi:10.1186/s12284-015-0058-1)
Supplement: Additional file 7: Table S1. — Primers for RT-qPCR. (DOCX 22 kb) [file 12284_2015_58_MOESM7_ESM.docx]

**Additional file 7: Table S1. Primers for RT-qPCR.**

| **Gene** | **Forward (5' - 3')** | **Reverse (5' - 3')** |
| --- | --- | --- |
| *GAMYB* | CAGTGGCAATTCATTCACTGAATC | TCCAGATCCCATTGAAGTGCTTTG |
| *CYP703A3* | GCTAGGGAGGCCAAGAAGAG | TTGGTCACCGATGATGTGTC |
| *KAR* | ACATGACCGCAAAACTAGGC | ATTGACAGGCCACCATCAAC |
| *UBQ5* | ACCACTTCGACCGCCACTACT | ACGCCTAAGCCTGCTGGTT |
